# Dissociated modulations of multivoxel activation patterns in the ventral and dorsal visual pathways by the temporal dynamics of stimuli

**DOI:** 10.1002/brb3.1673

**Published:** 2020-06-04

**Authors:** Jiaxin Li, Bingbing Guo, Lin Cui, Hong Huang, Ming Meng

**Affiliations:** ^1^ School of Psychology South China Normal University Guangzhou China; ^2^ Key Laboratory of Brain Cognition and Education Sciences (South China Normal University) Ministry of Education Guangzhou China; ^3^ Center for Studies of Psychological Application South China Normal University Guangzhou China; ^4^ Guangdong Key Laboratory of Mental Health and Cognitive Science South China Normal University Guangzhou China

**Keywords:** face perception, fMRI, MVPA, temporal resolution, tool, visual pathways

## Abstract

**Introduction:**

Previous studies suggested temporal limitations of visual object identification in the ventral pathway. Moreover, multivoxel pattern analyses (MVPA) of fMRI activation have shown reliable encoding of various object categories including faces and tools in the ventral pathway. By contrast, the dorsal pathway is involved in reaching a target and grasping a tool, and quicker in processing the temporal dynamics of stimulus change. However, little is known about how activation patterns in both pathways may change according to the temporal dynamics of stimulus change.

**Methods:**

Here, we measured fMRI responses of two consecutive stimuli with varying interstimulus intervals (ISIs), and we compared how the two visual pathways respond to the dynamics of stimuli by using MVPA and information‐based searchlight mapping.

**Results:**

We found that the temporal dynamics of stimuli modulate responses of the two visual pathways in opposite directions. Specifically, slower temporal dynamics (longer ISIs) led to greater activity and better MVPA results in the ventral pathway. However, faster temporal dynamics (shorter ISIs) led to greater activity and better MVPA results in the dorsal pathway.

**Conclusions:**

These results are the first to show how temporal dynamics of stimulus change modulated multivoxel fMRI activation pattern change. And such temporal dynamic response function in different ROIs along the two visual pathways may shed lights on understanding functional relationship and organization of these ROIs.

## Graphical abstract

By combining time‐resolved fMRI and multivariate pattern analysis (MVPA), here we reveal opposite modulations of stimulus temporal dynamics in the ventral and dorsal visual pathways for fMRI response patterns as a function of interstimulus interval (ISI). The results support more rapid temporal encoding in the dorsal pathway than the ventral pathway, shedding lights for understanding functional relationship and organization of the two visual pathways.

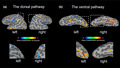

## 1 INTRODUCTION

It is currently a popular assumption that the visual pathway consisted of two distinct pathways. The ventral pathway is involved in object identification, projecting from the primary visual cortex (V1) to the inferior temporal lobe (Ungerleider & Mishkin, 1982). By contrast, the dorsal pathway projecting from V1 to the posterior parietal lobe is concerned with visually guided action, such as reaching a target and grasping a tool (Goodale & Milner, 1992). Consistent with this notion, through fMRI measurements of BOLD activity, a host of object selective areas are reported in the ventral pathway. For example, an area in the inferior temporal lobe has been hypothesized to involve in face perception, selectively responsive to face images (the fusiform face area, FFA) (Fairhall & Ishai, 2006; Grill‐Spector, Knouf, & Kanwisher, 2004; Grill‐Spector & Weiner, 2014; Grill‐Spector, Weiner, Gomez, Stigliani, & Natu, 2018; Gschwind, Pourtois, Schwartz, Van De Ville, & Vuilleumier, 2011; Guo & Meng, 2015; Kanwisher, McDermott, & Chun, 1997; Saygin et al., 2012), whereas the superior parietal lobule (SPL) in the posterior parietal lobe has been hypothesized to involve in action control, responding to tool images more strongly than to nontool images (Brandi, Wohlschläger, Sorg, & Hermsdörfer, 2014; Chao & Martin, 2000; Chen, Snow, Culham, & Goodale, 2017; Culham & Valyear, 2006; Hermsdörfer, Terlinden, Mühlau, Goldenberg, & Wohlschläger, 2007; Lewis, 2006; Mruczek, von Loga, & Kastner, 2013; Peeters et al., 2009).

However, relationships between the two pathways have also been proposed. For example, implied motion is perceived when observers have recognized animate objects in static pictures (Kourtzi & Kanwisher, 2000; Lorteije et al., 2006). As the dorsal pathway typically processes motion information to guide action, the perception of implied motion would involve both the ventral and dorsal pathways. Indeed, visual implied motion was found to be encoded in the dorsal pathway, suggesting dynamic interactions between the two visual pathways (Lu, Li, & Meng, 2016). Similarly, object recognition sometimes rely on perceiving structure from motion (Kourtzi, Krekelberg, & Van Wezel, 2008; Murray, Olshausen, & Woods, 2003). Several studies found brain regions in both the ventral and dorsal pathways involved in structure from motion processing (Kourtzi, Bülthoff, Erb, & Grodd, 2002; Paradis et al., 2000; Wang et al., 1999). The integration of structure recognition and motion processing again reflects functional interactions between the two visual pathways. Taken together, many studies suggest the two pathways may closely interact and consist with the “vision‐for‐perception” and “vision‐for‐action” networks (Almeida, Fintzi, & Mahon, 2013; Bracci & Beeck, 2015; Freud, Culham, Plaut, & Behrmann, 2017; Freud, Plaut, & Behrmann, 2016; Garcea, Kristensen, Almeida, & Mahon, 2016; Kristensen, Garcea, Mahon, & Almeida, 2016; Mahon, Kumar, & Almeida, 2013; Chen, Garcea, Almeida, & Mahon, 2017).

Even if there were interactions between the two visual pathways, temporal dynamics of the interactions are unknown. Several studies suggested more rapid processing in the dorsal pathway than the ventral pathway, as that responses to high temporal dynamic visual stimuli were found primarily in the dorsal pathway (Kristensen et al., 2016; Liu & Wandell, 2005; Stigliani, Jeska, & Grill‐Spector, 2017), and that perceptual integration may be formed quickly in the dorsal pathway (Liu, Wang, Zhou, Ding, & Luo, 2017). By contrast, studies of implied motion suggested ventral pathway process “what” information first, indicating that temporal processing in the ventral pathway would be faster than the dorsal pathway. Moreover, a few fMRI studies estimated how much information can be processed in a unit of time in the two visual pathways. For example, the univariate averaged BOLD response of FFA peaked at the temporal rate of 4–5 items per second, suggesting a capacity limit of temporal processing (McKeeff, Remus, & Tong, 2007; Stigliani, Weiner, & Grill‐Spector, 2015). While another recent fMRI study examined how brain activity in the dorsal pathway would be modulated by temporal frequency of stimuli, relationship between the two visual pathways in capacity limit of temporal processing remains largely unclear (Kristensen et al., 2016; Liu & Wandell, 2005; Stigliani et al., 2017).

Here, fMRI activity corresponding to watching images of faces and t·ools was measured in our study to examine the temporal processing capacities in the brain areas within two visual pathways (e.g., FFA and SPL). Different from previous fMRI studies that only analyzed univariate averaged BOLD responses to investigate the temporal capacity, multivoxel pattern analysis (MVPA) was employed in our study. Comparing to MVPA, univariate analysis may poorly reveal object category encoding (Chen, Garcea et al., 2017; Guo & Meng, 2015). Multivoxel activity patterns are also known to comprise faster temporal dynamics than univariate averaged BOLD responses (Kohler et al., 2013).

In addition, motivated by time‐resolved papers (Carlson, Grol, & Verstraten, 2006; Dux, Jason, Asplund, & René, 2006; Formisano & Goebel, 2003; Ogawa et al., 2000), we examined the modulation of temporal dynamics of stimuli by manipulation of interval between two stimulus images. By repeatedly sampling brain activity while participants repeatedly performed a task with temporal jitter, we were able to discern the duration of a neurophysiological process. For example, the dynamic neural basis underlying dual‐task limitation was investigated by using two stimulus‐onset‐asynchronies (SOAs): The SOA between the two tasks was either 300 ms or 1,560 ms (Dux et al., 2006). It was hypothesized that the two tasks would interfere more for the short SOA condition than for the long SOA condition. According to increasingly longer response time to the second task as the SOA decreases, it was then deducted that the responses of brain regions, whose temporal profile of activation tracked the time course of dual‐task processing, should be modulated by the varying SOA. Consistent with this notion, a neural network of frontal lobe areas was found to be a temporal processing bottleneck for multitasking (Dux et al., 2006). Similarly, temporal dynamics of inferotemporal cortex activity in visual object recognition (Carlson et al., 2006), posterior parietal cortex activity in mental imagery (Formisano et al., 2002), primary visual area activity in flash visual stimulation (Ogawa et al., 2000) were effectively estimated. Closely related to this idea, rapid serial visualpresentation (RSVP) has been used to estimate the rate at which the visual system can process a series of objects (McKeeff et al., 2007; Robinson, Grootswagers, & Carlson, 2019; Stigliani et al., 2015).

Specifically, we investigated fMRI responses corresponding to participants watching two stimulus images that were serially presented. The interstimulus interval (ISI) between the first and second stimulus images varied at four levels (33, 67, 133, and 267 ms). Both univariate analysis and MVPA were conducted to evaluate the effect of ISI in the FFA (ventral pathway) and SPL (dorsal pathway). Results of previous studies suggested that the capacity limit of temporal processing in the FFA is about 4–5 items per second (McKeeff et al., 2007; Stigliani et al., 2015). If capacity limit of temporal processing in the SPL would be faster than the FFA, we may find responses in the SPL peak at shorter ISIs (i.e., 33 or 67 ms) than at longer ISIs (i.e. 133 or 267 ms). However, if we would find responses in the SPL peak at a similar rate to the FFA, it would suggest no dissociation of the capacity limit of temporal processing in the two visual pathways. As the FFA and SPL were localized on the basis of object category selectivity (faces vs. tools), to clarify that there are no category selectivity and two‐pathway confounding, additionally we performed searchlight mapping to identify brain regions in which responses decreased/increased as a function of ISI.

## 2 MATERIALS AND METHODS

### 2.1 Participants

Eighteen right‐handed participants (8 male; ages 20–40) with normal or corrected to normal visual acuity participated in the experiment. Data of one participant were excluded from further analyses due to anatomical abnormalities revealed by structural MRI. The study was approved by human subjects review committee of South China Normal University. All participants provided written informed consent.

### 2.2 MRI data acquisition

MRI scanning was performed on 3‐T Siemens Trio with a 32‐channel head coil at the Brain Imaging Center of South China Normal University. For each participant, a high‐resolution 3D magnetization‐prepared rapid acquisition gradient echo (MPRAGE) structural scan was acquired (TR = 2,300 ms, TE = 3.24 ms, flip angle = 9°, FOV = 256 × 256 mm^2^, voxel size = 1 × 1 × 1 mm^3^, 176 slices). BOLD signals were acquired with an echo planar imaging (EPI) sequence (TR = 2,000 ms, TE = 30 ms, flip angle = 90°, FOV = 192 × 192 mm^2^, voxel size = 3 × 3 × 3 mm^3^, 32 slices). In the scanner, visual stimuli were presented via a LCD projector (60 Hz refresh rate, 1,024 × 768 pixel resolution) using MATLAB (The Math‐Works) and Psychophysics Toolbox (Brainard, 1997).

### 2.3 Procedures and experimental design

#### 2.3.1 Functional localizer

Regions of interest (ROIs) in both visual pathways were functionally localized with separate scan runs by contrasting brain activation corresponding to an independent set of faces images versus tools images that were not used in the main experimental runs. These ROIs include the FFA and SPL that were preidentified according to literatures, to avoid "double‐dipping" analyses (Nikolaus, Kyle, Bellgowan, & Baker, 2009). To localize the functional ROIs, each participant was asked to complete two 336 s localizer runs. Each run consisted of 10 stimulus blocks interleaved with 11 fixation blocks. The stimulus blocks consisted of five face‐image blocks and five tool‐image blocks that were presented in a random order. In each stimulus block, there were 16 visual stimuli, and each visual stimulus was presented at the center of screen for 500 ms, followed by a 500 ms fixation‐only interval. Four of the stimulus images in each block may be presented repeatedly. To ensure that participants attended to the stimuli, they were asked to report whether each presented image had been new.

#### 2.3.2 Main experiment

**FIGURE 1:**
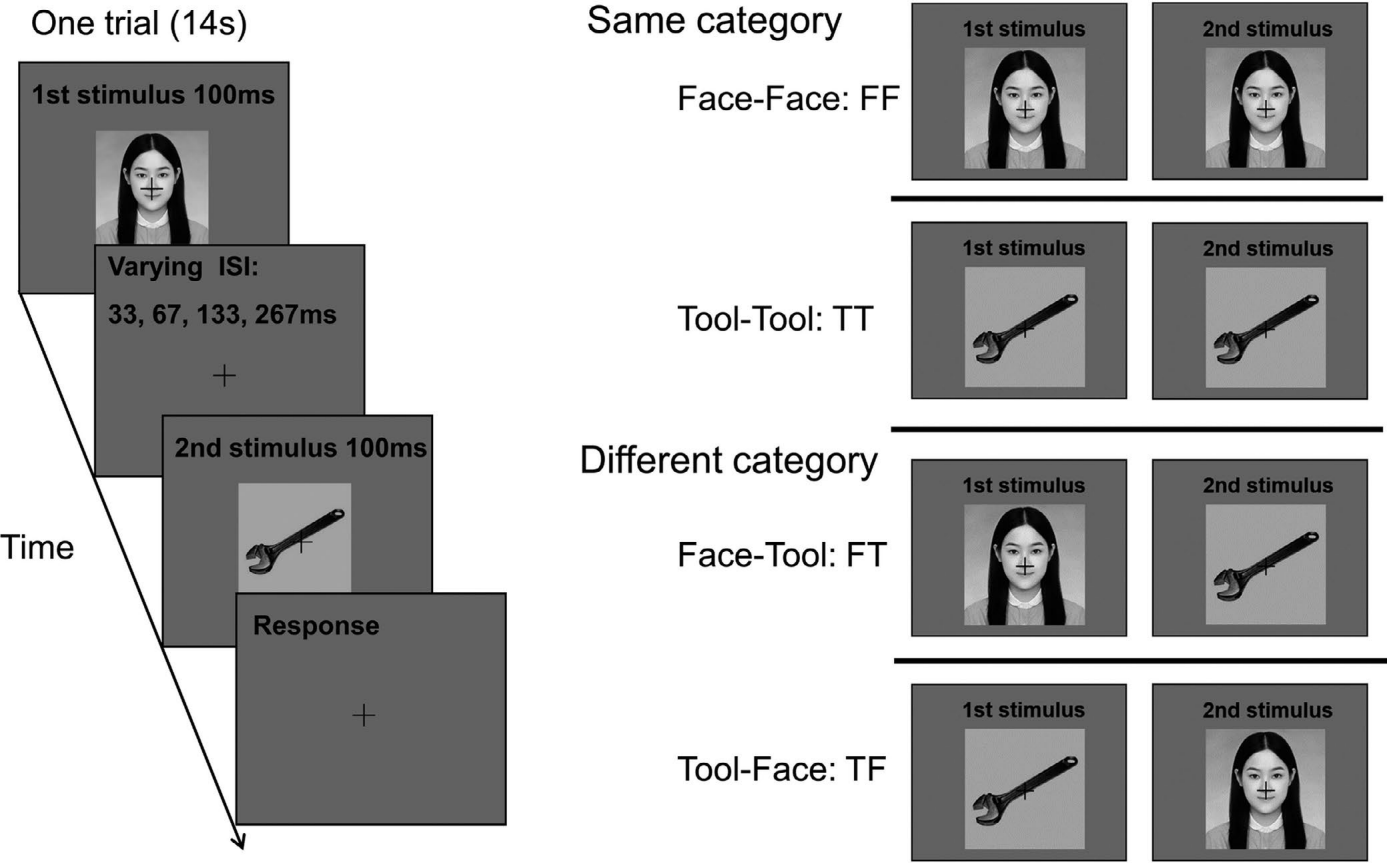
Slow event‐related experimental design. Each trial was 14s long. Left: In each trial, the stimuli were presented for 100 ms per image in succession. Critically, the ISI between the first and second stimulus varied at four levels (33, 67, 133, and 267 ms). Right: Four main stimulus conditions with the successionally presented two stimulus images belonging to either the same category (FF or TT) or different categories (FT or TF)

Each participant performed ten main experiment runs. A slow event‐related design was used. There were four experimental conditions: (a) a face was shown the first followed by a face (Face–Face: FF); (b) a tool was shown the first followed by a tool (Tool–Tool: TT); (c) a face was shown the first followed by a tool (Face‐Tool: FT); (d) a tool was shown the first followed by a face (Tool‐Face: TF). In each trial, two 100 ms images were presented in quick succession, shown in Figure 1. Participants were asked to report whether the second stimulus image of each trial was a face or a tool. Critically, the ISI between the first and second image varied at four levels (33, 67, 133, and 267 ms). Thus, in total, each run consisted of 16 experiment trials (4 conditions × 4 ISIs). A blank display with a fixation that was presented at the center of the screen was shown after the second stimulus image, to make each trial 14 s long, and each run began with a 14 s period of such fixation‐only display.

### 2.4 Data analysis

#### 2.4.1 Preprocessing

Preprocessing was conducted by using AFNI (Cox, 1996). All EPIs were head movements corrected, spatially smoothed with a 4 mm full width at half maximum (FWHM), filter and linear drift corrected to remove baseline drifts. Slice timing correction was conducted for the main experimental EPIs. All data were then transformed according to the Talairach template into normalized coordinates (Talairach & Tournoux, 1988).

#### 2.4.2 ROIs localization

To define functional ROIs, a whole‐brain general linear model (GLM) analysis was performed. The right FFA in 15 out of the 17 participants was individually localized as a cluster of 20 or more contagious voxels that show significantly stronger activation for faces than for tools (*p* < 10^–3^, uncorrected) in the right fusiform gyrus (Kanwisher et al., 1997). The right FFA for the other two participants was defined by comparing the face‐image blocks with fixation blocks (*p* < 10^–30^, uncorrected, cluster size >20 voxels). Similarly, the left SPL in 11 participants was individually localized as a cluster of 20 or more contagious voxels that show significantly stronger activation for tools than for faces (*p* < 10^–3^, uncorrected) (Chao & Martin, 2000; Kristensen et al., 2016). The left SPL for the other six participants was defined by comparing the tool‐image blocks with fixation blocks (*p* < 10^–7^, uncorrected, cluster size >20 voxels). The mean Talairach coordinates of right FFA were [+39.41 ± 0.91, −50.87 ± 1.58, −17.63 ± 0.65] in LPI coordinates, and those of left SPL were [−22.61 ± 1.28, −64.16 ± 1.97, +51.86 ± 1.24] in LPI coordinates. In comparison, we also analyzed an ROI in the early visual cortex, Brodmann area 17 (BA17), which was localized by using an anatomical mask (TT_N27 template). Through this procedure, the BA17 was localized in all 17 participants, and the Talairach coordinates of the localized BA17 were [+0.5, −87.1, +5.6] in LPI coordinates.

#### 2.4.3 Univariate analysis

The time courses of BOLD signals were extracted by averaging percent signal change (PSC) across all voxels in each ROI. To calculate the PSC of each trial, baseline was defined as averaged of activity at the last TR before and the first TR of trial onset. Consistent with previous studies, the PSC peaked at the third TR after stimuli onset (Aguirre, Zarahn, & D'esposito, M., 1998; Lu et al., 2016; Miezin, Maccotta, Ollinger, Petersen, & Buckner, 2000). The PSC peak at the third TR was then used in subsequent multivariate analysis.

#### 2.4.4 Multivariate pattern analysis

Multivariate pattern analysis (MVPA) was performed by using PyMVPA (Hanke et al., 2009) and the PSC peak values of all trials were employed. Through a leave‐one‐trial‐out cross‐validation procedure, pattern classification of the FF versus TT (same category) was performed with linear support vector machines (SVMs). Prediction of each trial in FF and TT conditions was exported from the classification as FF or TT and then was used to calculate classification accuracy for these two conditions of four ISIs. The same analysis was also conducted for different category conditions (FT and TF).

#### 2.4.5 Multivariate searchlight analysis

A whole‐brain searchlight analysis was employed by using PyMVPA and MATLAB (Kriegeskorte, Goebel, & Bandettini, 2006). For each participant, activity patterns were extracted from a spherical searchlight with a two‐voxel radius (33 voxels in each searchlight including the central voxel) that traversed all gray matter voxels. Then, MVPA was performed by using linear SVMs for each searchlight ROI corresponding to a central voxel (i.e., each voxel across the whole gray matter mask). To ensure independence between training and testing, cross‐validations were performed using the leave‐one‐trial‐out procedure, and then classification accuracy of the FF versus TT conditions was calculated. To further understand the dissociated modulations of the activation patterns between the two visual pathways, planned linear trend analyses of the effect of ISI were conducted. Then, slope of the linear trend for classification accuracy as a function of ISI was calculated for each participant. After spatially smoothing (4 mm FWHM), statistical analysis (*t* test) across all participants was performed for each voxel. Finally, the slope for each searchlight ROI was mapped by using SUMA (AFNI surface mapper; Saad & Reynolds, 2012).

## 3 RESULTS

All participants (*n* = 17) maintained high‐performance accuracy on the behavioral task (mean = 97.79 ± 0.51%) during scanning. Firstly, the influence of ISI on response accuracy was small, based on response accuracy, ANOVAs were performed to evaluate the effects of stimulus condition (FF vs. TT) and ISI (33, 67, 133, 267 ms). The main effect of stimulus condition was significant (*F*_(1,16)_ = 5.474, *p* < .05), while the main effect of ISI and the interaction effect were not significant (*Fs* < 0.136, *n.s*.). Secondly, for reaction times, the main effect of stimulus condition was marginally significant (*F*_(1,16)_ = 4.386, *p* = .053, $\eta_{p}^{2}$ = 0.215), the main effect of ISI was significant (*F*_(3,48)_ = 5.543, *p* < .01, $\eta_{p}^{2}$ = 0.257), and the interaction was also significant (*F*_(3,48)_ = 4.876, *p* < .01, $\eta_{p}^{2}$ = 0.234). Further one‐way ANOVAs suggested that the effect of ISI was not significant (*F*_(3,48)_ = 1.692, *p* > .05, $\eta_{p}^{2}$ = 0.096) in the FF condition, while the effect of ISI was highly significant (*F*_(3,48)_ = 9.122, *p* < 10^–4^, $\eta_{p}^{2}$ = 0.363) in the TT condition. These results may merely indicate that our behavioral task is too easy (accuracy >95%) and easier as ISI increases (reaction time decreases with longer ISIs).

### 3.1 Univariate averaged BOLD activity

**FIGURE 2:**
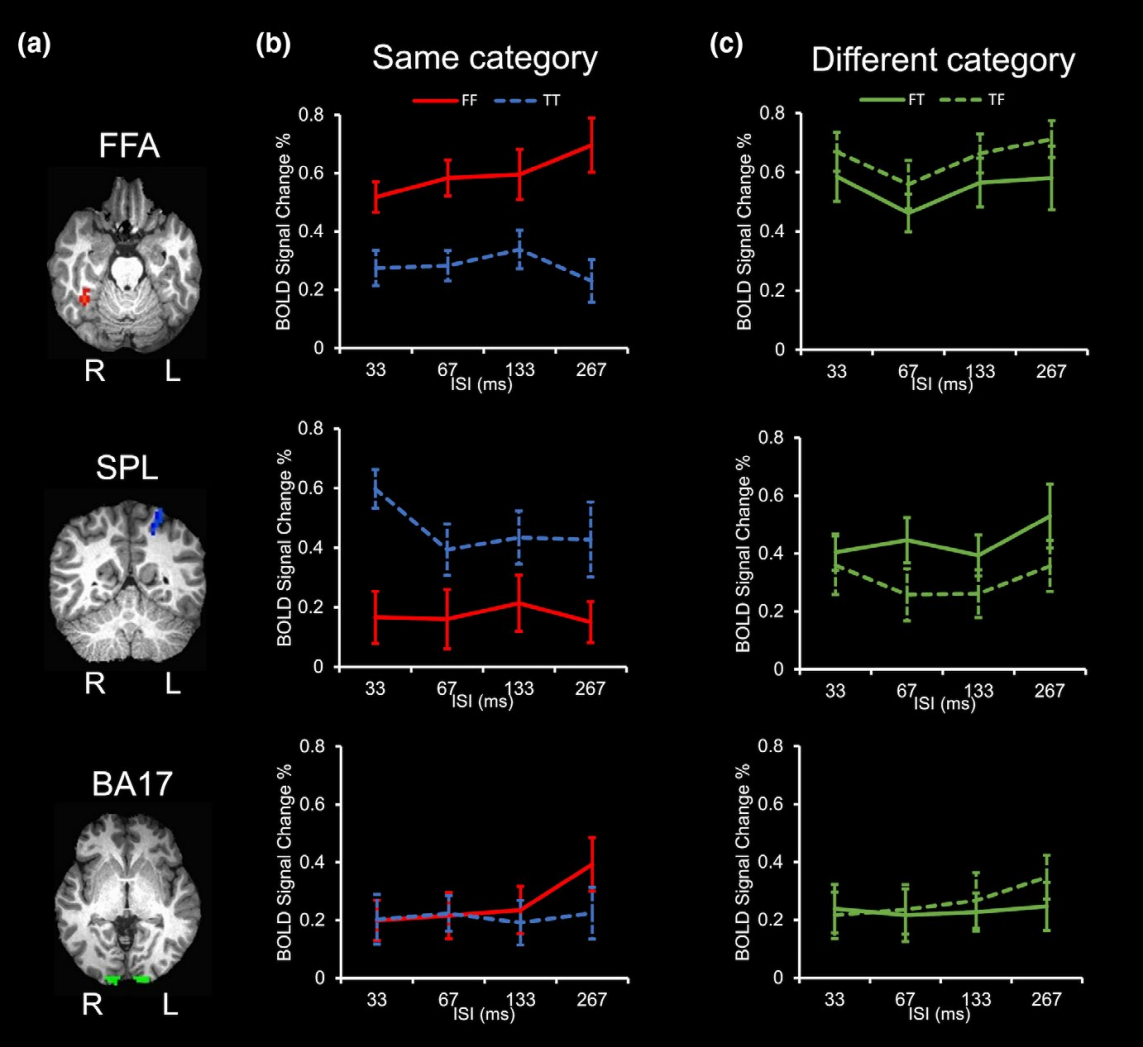
Results of univariate averaged BOLD responses. (a) The localization of regions of interest depicted on the brain of a representative participant. (b) Effects of ISI on averaged BOLD responses of the FFA, SPL, and BA17 corresponding to when the first and second stimulus image belonged to the same category (red solid lines: the FF condition; blue dash lines: the TT condition). (c) Effects of ISI on averaged BOLD responses corresponding to when the first and second stimulus image belonged to different categories (solid lines: the FT condition; dash lines: the TF condition). Error bars represent 1 *SEM*. BA17, Brodmann area 17; FFA, fusiform face area; FF, Face–Face; FT, Face‐Tool; L, left hemisphere; R, right hemisphere; SPL, superior parietal lobule; TF, Tool‐Face; TT, Tool–Tool

To evaluate whether the results described above were driven by averaged BOLD responses of the ROIs, conventional univariate fMRI analyses were conducted for the averaged amplitudes of event‐related BOLD PSC that were extracted from the FFA, SPL, and BA17, respectively. Results of the BOLD PSC as a function of ISI are shown in Figure 2. The averaged BOLD activity of FF in the FFA increased as a function of ISI, as the fMRI responses to larger ISI (267 ms) was the peak activity. While the averaged BOLD activity of TT in the SPL decreased as a function of ISI, as the fMRI responses to shorter ISI (33 ms) was the peak activity. Firstly, we conducted a three‐way ANOVAs to evaluate the effects of ROI (right FFA vs. left SPL), ISI (33, 67, 133, 267 ms), and category (FF vs. TT). The interaction between ROI and category was highly significant (*F*_(1,32)_ = 41.439, *p* < 10^–6^, $\eta_{p}^{2}$ = 0.564), and the main effect of ROI was marginally significant (*F*_(1,32)_ = 3,452, *p* = .072, $\eta_{p}^{2}$ = 0.097), while all other effects and interactions were not significant (*Fs* < 1.096, *n.s*.). These results merely replicate that the FFA responded strongly to the FF condition whereas the SPL responded strongly to the TT condition.

For the same category conditions, two‐way ANOVAs were conducted to compare the FF versus TT conditions for each ROI. The main effect of condition was significant in the FFA (*F*_(1,16)_ = 27.257, *p* < 10^–5^, $\eta_{p}^{2}$ = 0.630) and in the SPL (*F*_(1,16)_ = 14.602, *p* < .01, $\eta_{p}^{2}$ = 0.477), but not in the BA17 (*F*_(1,16)_ = 2.119, *p* > .05, $\eta_{p}^{2}$ = 0.117). All other effects and interactions were not significant (*Fs* < 2.308, *n.s*.). For the different categories (FT and TF) conditions, the main effect of condition was significant in the FFA (*F*_(1,16)_ = 14.629, *p* < .001, $\eta_{p}^{2}$ = 0.478), but not in the SPL (*F*_(1,16)_ = 0.812, *p* > .05, $\eta_{p}^{2}$ = 0.048) and in the BA17 (*F*_(1,16)_ = 1.139, *p* > .05, $\eta_{p}^{2}$ = 0.066). The main effect of ISI was significant in the FFA (*F*_(3,48)_ = 4.488, *p* < .01, $\eta_{p}^{2}$ = 0.219) and in the SPL (*F*_(3,48)_ = 2.961, *p* < .05, $\eta_{p}^{2}$ = 0.156). All other effects and two‐way interactions were not significant (*Fs* < 1.094, *n.s*.). These results suggest that univariate averaged BOLD activity alone could not reveal to a fine scale for examining the temporal processing capacity of the ROIs along the two visual pathways.

### 3.2 Faster temporal processing capacity in the SPL than the FFA revealed by MVPA

**FIGURE 3:**
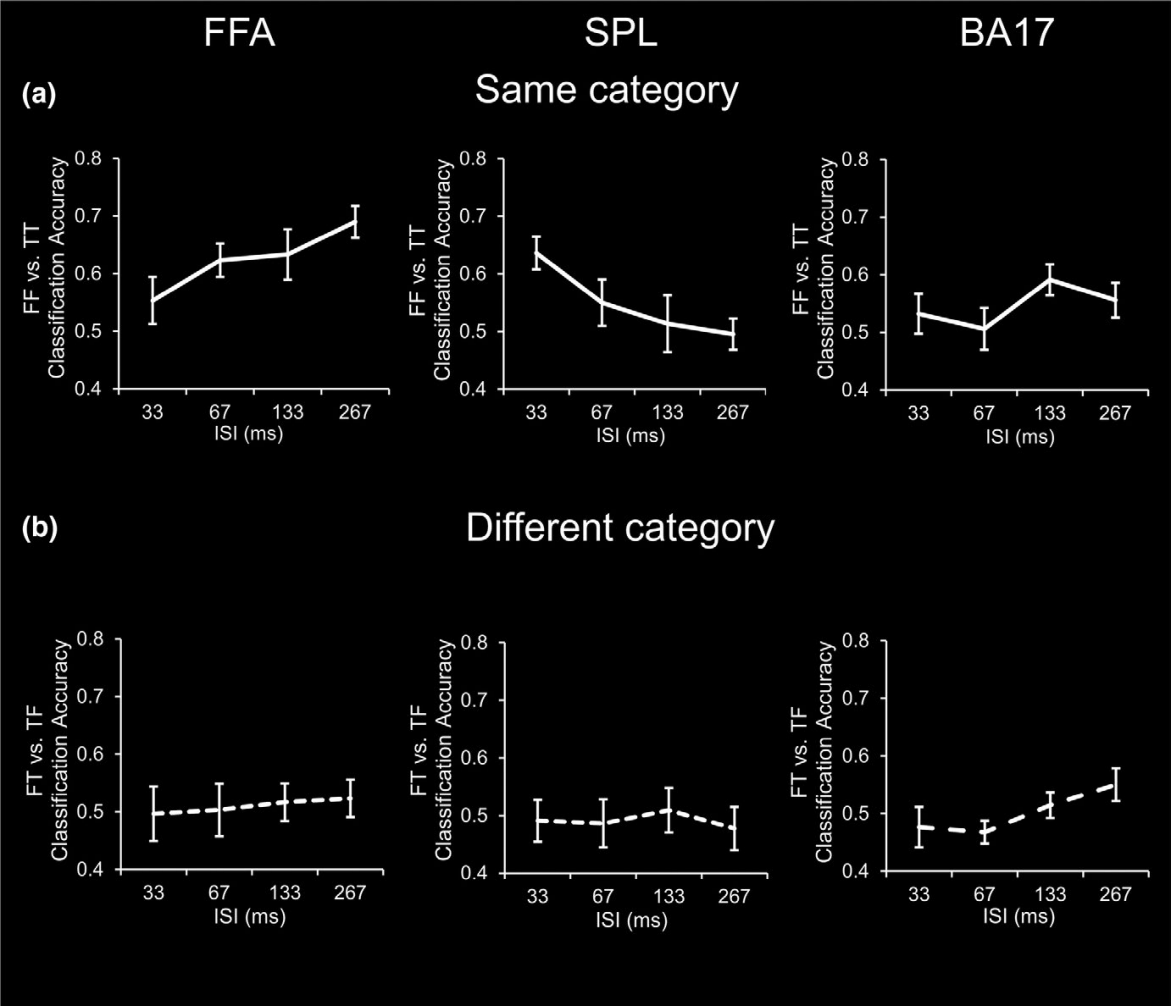
Results of MVPA in the FFA, SPL, and BA17. (a) Activity pattern classification accuracy of FF versus TT conditions as a function of ISI (solid lines). (b) Activity pattern classification accuracy of FT versus TF conditions as a function of ISI (dash lines). Error bars represent 1 *SEM*. BA17, Brodmann area 17; FFA, fusiform face area; FF, Face–Face; FT, Face‐Tool; MVPA, multivoxel pattern analyses; SPL, superior parietal lobule; TF, Tool‐Face; TT, Tool–Tool

The ROI‐based MVPA results are shown in Figure 3. In the FFA (ventral), classification accuracy of the FF versus TT conditions significantly increased as a function of ISI (*F*_(3,48)_ = 3.171, *p < *.05, $\eta_{p}^{2}$ = 0.165). By contrast, in the SPL (dorsal), classification accuracy of the FF versus TT conditions significantly decreased as a function of ISI (*F*_(3,48)_ = 3.496, *p < *.05, $\eta_{p}^{2}$ = 0.179). These results suggest that the maximum temporal processing capacity of the FFA would be 367ms (267 ms ISI plus 100 ms stimulus display) or longer and that in the SPL would be 133 ms (33 ms ISI plus 100 ms stimulus display) or shorter. For the BA17, the effect of ISI was not significant (*F*_(3,48)_ = 2.744, *p* > .05, $\eta_{p}^{2}$ = 0.146), suggesting that our results may not be driven by low‐level stimulus properties. For comparisons, classification accuracy of the FT versus TF conditions was neither significantly above the chance level (*ts* < 1.758, *ps* > 0.098), nor modulated by ISI (*Fs* < 1.761, *ps* > 0.167). Presumably, sluggish BOLD signal would lead to temporal mixing of the responses to face and tool stimuli. Therefore, the classification accuracy of the FT versus TF conditions failed to reach statistical significance. However, previous studies suggested that temporal processing capacity could be assessed by BOLD signals corresponding to RSVP stimuli that belonged to a same category (McKeeff et al., 2007; Robinson et al., 2019; Stigliani et al., 2015). Consistent with this notion, when the first and second stimulus image belonged to the same category (FF and TT conditions) in our study, the temporal relations between the first and second stimulus image modulated fMRI activity, as shown by the significant effects of ISI in the FFA and SPL.

For the same category conditions, to further specifically compare the FFA and SPL that represent the two visual pathways respectively, we conducted within‐subject model repeated‐measure ANOVAs to evaluate the effects of ROI (FFA vs. SPL) and ISI (33, 67, 133, 267 ms). The main effect of ROI was significant (*F*_(1,16)_ = 6.828, *p* < .05, $\eta_{p}^{2}$ = 0.299), while the main effect of ISI was not significant (*F*_(3,48)_ = 0.472, *p* > .05, $\eta_{p}^{2}$ = 0.029). Critically, the interaction between ROI and ISI was significant (*F*_(3,48)_ = 5.795 *p* < .01, $\eta_{p}^{2}$ = 0.266), suggesting a marked dissociation of temporal processing capacity between the FFA and SPL.

### 3.3 Dissociation of temporal processing capacity along the two visual pathways revealed by results in other ROIs and multivariate searchlight analysis

Given that the most interesting findings of the present study were in the right FFA and left SPL, one may wonder what about other ROIs in the two visual pathways. To address this issue, using the same methods to localize the FFA (faces > tools), we were able to functionally localize the STS (Superior temporal sulcus) in all participants (*N* = 17), the OFA (Occipital face area) in 12 out of the 17 participants. We also localized fusiform tool area and IPL (Inferior parietal lobule) in all participants as how we localized the SPL (tools > faces). The ventral ROIs included FFA, OFA, STS, and fusiform tool area. The dorsal ROIs mainly consisted of the SPL and IPL. Similar to the FFA, the MVPA decoding accuracies of FF versus TT of the bilateral OFAs increased as a function of ISI. Further one‐way ANOVAs suggested that the effect of ISI was marginally significant (in the right OFA: *F*_(3,33)_ = 2.435, *p* = .082, $\eta_{p}^{2}$ = 0.181; in the left OFA: *F*_(3,33)_ = 2.440, *p* = .082, $\eta_{p}^{2}$ = 0.182). Although the ISI effect was not significant in the STS and fusiform tool area, results in the FFA and OFA suggested similar activation patterns (accuracies increased as a function of ISI) in brain areas in the ventral pathway. The dorsal ROIs mainly consisted of the SPL and IPL. The MVPA decoding accuracies in the SPL decreased as a function of ISI, but the ISI effect was not significant in the IPL. Specifically, two‐way ANOVAs of ROI (SPL/IPL) × ISI (33, 67, 133, 267ms) showed no significant main effect of ROI (*F*_(1,16)_ = 2.057, *p* > .05, $\eta_{p}^{2}$ = 0.114). One might argue there were categories and pathways confounding since the FFA and OFA (ventral ROIs) responded stronger to faces, while the SPL and IPL (dorsal ROIs) responded stronger to tools. Thus, we conducted a linear trend analysis on the decoding accuracy of fusiform tool area (respond stronger to tools) in the ventral pathway. The positive linear trend indicated the accuracies increased as a function of ISI in the fusiform tool area, different from results in the SPL/IPL (respond stronger to tools) in the dorsal pathway. Therefore, we think it is unlikely that there were categories and pathways confounding.

**FIGURE 4:**
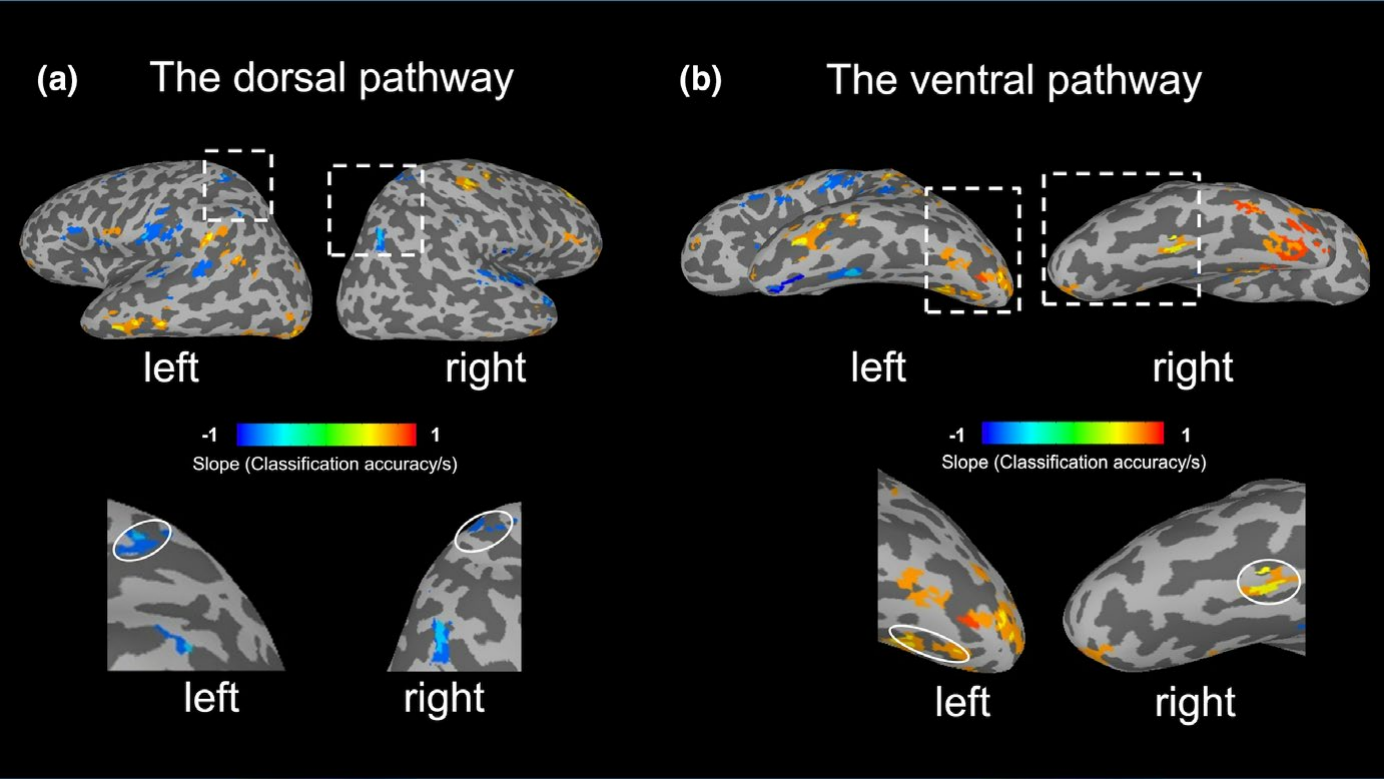
Results of searchlight analysis. Linear trends for classification accuracy (FF vs. TT) as a function of interstimulus interval (ISI) were estimated based on activity patterns that were extracted from a 33‐voxel spherical searchlight that traversed all gray matter voxels. (a), Significant negative linear trends were found in the dorsal pathway, and the Talairach coordinates of regions of interest (ROIs) for the left and right hemispheres were [−25.4, −39.9, +52.1] and [+37.5, −72.3, +43.9] in LPI coordinates, respectively. (b), Significant positive linear trends were found mainly in the ventral pathway, and the Talairach coordinates of ROIs for the left and right hemispheres were [−16.3, −76.3, −8.9] and [+36.9, −51.2, −21.1] in LPI coordinates, respectively. Color scale represents the slope of significant linear trends (*p* < .01) for classification accuracy as a function of ISI. FF, Face–Face; TT, Tool–Tool

To further explore the temporal dynamics of object processing across the ventral and dorsal pathways, a multivariate searchlight analysis was performed on the main experiment data. It is worth noting that, the localizations of the FFA/SPL were independent from the searchlight analysis, and we had decided to select these ROIs before the searchlight analysis, thus our selection of ROIs was not biased by the searchlight results. Figure 4 shows the map of brain areas with significant (*p* < .01) linear trends for the classification accuracy as a function of ISI. The dissociation between the two visual pathways is evident. Significant negative linear trends (blue colors) were found in the dorsal pathway, whereas significant positive linear trends (orange and yellow colors) were found mainly in the ventral pathway. In addition, clusters (cluster size >40 voxels) with significant linear trends (*p* < .01) in the ventral and dorsal pathways are presented in Tables 1 and 2, respectively. Seven ROIs with significant positive linear trends were found in the ventral pathway, and four ROIs with significant negative linear trends were found in the dorsal pathway. Taken together, these results suggest that the dorsal pathway would process rapidly presented stimuli more efficiently than slowly presented stimuli, whereas the ventral pathway would be the opposite and therefore slower than the dorsal pathway for processing the stimuli.

**TABLE 1:** Regions with significant positive linear trends for the classification accuracy as a function of ISI in the ventral pathway

Hemisphere	Location	Number of Voxels	Peak *x*	Peak *y*	Peak *z*	Slope (Classification/s)
Right	Cerebellum (Temporal lobe)	218	22.5	−31.5	−36.5	0.334
Left	Fusiform Gyrus	197	−22.5	−82.5	−9.5	0.372
Left	Lingual Gyrus	102	−16.5	−61.5	−6.5	0.366
Right	Lingual Gyrus	53	1.5	−70.5	−0.5	0.325
Left	Inferior Temporal Gyrus	52	−55.5	−22.5	−15.5	0.312
Right	Cerebellum (Temporal lobe)	50	46.5	−64.5	−24.5	0.223
Right	Fusiform Gyrus	48	37.5	−49.5	−21.5	0.362

**TABLE 2:** Regions with significant negative linear trends for the classification accuracy as a function of ISI in the dorsal pathway

Hemisphere	Location	Number of Voxels	Peak x	Peak y	Peak z	Slope (Classification/s)
Right	Posterior Cingulate Gyrus	139	4.5	−25.5	23.5	−0.314
Right	Superior Parietal Lobule	136	37.5	−70.5	44.5	−0.391
Left	Postcentral Gyrus	108	−58.5	−16.5	14.5	−0.333
Left	Inferior Parietal Lobule	57	−43.5	−64.5	44.5	−0.338

## 4 DISUSSION

We used MVPA and ISI manipulation to overcome temporal delay of BOLD responses, and compared how the two visual pathways respond to the dynamics of visual stimuli. The MVPA results suggest that the temporal dynamics of stimuli led to dissociated modulations of activation patterns in the two pathways. Specifically, shorter ISIs (33 ms, 67 ms) led to better decodability for FF versus TT conditions in the dorsal pathway. By contrast, longer ISIs (133 ms, 267 ms) led to better decodability for the FF versus TT conditions in the ventral pathway. In comparison, the effect of ISI was not significant for decoding the FT versus TF conditions, confirms sluggish BOLD responses and that our results were not driven by any artifacts due to the variation of temporal presentation for the second stimuli. Instead, our time‐resolved approach revealed only the dynamic interaction between repeatedly presented stimuli.

Previous studies suggested temporal limitation of object processing capacity in the ventral pathway by using RSVP (Gauthier, Eger, Hesselmann, Giraud, & Kleinschmidt, 2012; McKeeff et al., 2007; Stigliani et al., 2015). For example, face‐selective areas and place‐selective areas showed peak tuning at about 4–5 items per second. Consistent with the notion of limited temporal capacity in the ventral pathway, electrophysiological studies have revealed that neural responses were stronger for slower image presentation rates during RSVP (Keysers & Perrett, 2002; Keysers, Xiao, Földiák, & Perrett, 2001). However, limitation of temporal capacity in the dorsal pathway was unclear. Moreover, previous studies only analyzed univariate averaged BOLD responses to investigate the temporal processing capacity in the ventral pathway, while the univariate averaged BOLD responses are known to comprise slower temporal dynamics than MVPA (Kohler et al., 2013). Therefore, few studies were able to access the temporal processing capacity in the dorsal pathway, assuming it would have been much faster that the ventral pathway. Indeed, our results suggest that the dorsal pathway is most sensitive to temporal interactions between two rapidly presented stimuli for the shortest ISI (33 ms) we had tested. Future studies are needed to further examine how the dorsal pathway may respond to even faster stimuli with ISI shorter than 33 ms.

Why would the temporal processing capacity be faster in the dorsal pathway than in the ventral pathway? Previous studies suggested differential contributions of magnocellular (M) and parvocellular (P) cells to the two pathways (Ferrera, Nealey, & Maunsell, 1994; Mahon et al., 2013; Merigan & Maunsell, 1993; Stigliani et al., 2017). Specifically, it has been proposed that while the ventral pathway may receive both P and M inputs, the dorsal pathway may be largely biased to receive M inputs. Our results that dissociated modulations of fMRI patterns in the ventral and dorsal visual pathways by the temporal dynamics of stimuli, consist with a recent fMRI study that found fundamental temporal mechanisms that distinguish visual streams in the human brain (Stigliani, Jeska, & Grill‐Spector, 2019). Given the higher‐speed processing capacity of M cells than P cells, our results confirm the greater involvement of M cells in the dorsal pathway than in the ventral pathway. While the ventral pathway may receive both P and M inputs, limited contribution of the M inputs would not be enough for changing overall activation patterns in shorter ISI conditions in the ventral pathway. For comparisons, the FT and TF conditions may equally stimulate both the M and P cells over the period of ISIs that was examined in our experiment. Therefore, the effect of ISI was not significant for decoding the FT and TF conditions in our experiment. Different from the SPL and the FFA, responses in the BA17 were not modulated by ISI, suggesting that the effect of ISI may not be driven by other low‐level stimulus properties.

Given that the most interesting findings of the present study were in the right FFA and left SPL, we performed an additional analysis in which fMRI responses of the left and right hemispheres were combined. Results of the pooled bilateral FFA and SPL show MVPA decoding accuracies increase as a function of ISI in the FFA; while decrease as a function of ISI in the SPL. These results appear to be more consistent with different temporal property in visual object processing in the dorsal and ventral pathways, rather than in the right and left hemispheres.

Moreover, one may wonder what about other ROIs in the two visual pathways and argue categories and pathways confounding since the FFA (ventral ROI) respond stronger to faces, while the SPL (dorsal ROI) respond stronger to tools. To address this issue, we localized more brain areas using localizer data, the ventral ROIs included the FFA, OFA, STS, and fusiform tool area. The dorsal ROIs mainly consisted of the SPL and IPL. Although only marginally significant, results in the OFA largely replicated the results of the FFA. And results from the FFA and OFA (ventral pathway) versus the SPL and IPL (dorsal pathway) confirmed that response patterns are consistent within each pathway. To clarify that there were no categories and pathways confounding, we conducted a linear trend analysis on the decoding accuracy of the fusiform tool area (respond stronger to tools) in the ventral pathway. The positive linear trend indicated the accuracies increased as a function of ISI in the fusiform tool area, different from results in the SPL/IPL (respond stronger to tools) in the dorsal pathway.

Most importantly, our whole‐brain results (Figure 4) suggest that not only the left SPL, other four ROIs (Table 1) in the dorsal pathway would process rapidly presented stimuli more efficiently than slowly presented stimuli, whereas not only the right FFA, other seven ROIs (Table 2) in the ventral pathway would be the opposite and therefore slower than the dorsal pathway for processing the stimuli. It worth noting that, different from the FFA and SPL, there were no such significant category selective (faces vs. tools, *p* < 10^–4^) in these seven ROIs in the ventral pathway and in the four ROIs in the dorsal pathway. Therefore, it is further demonstrated that there were no categories and pathways confounding.

In summary, our study found that the temporal dynamics of stimuli led to dissociation of fMRI activation patterns in the two visual pathways. Given that activation patterns may reflect population responses of neuron ensembles, temporal encoding in the dorsal pathway appears to be faster than the ventral pathway. These findings may shed lights on further understanding functional relationship and organization of the two visual pathways. Methodologically, shortening the ISI practically enables us to assess the temporal profile of object category decoding, which has significant potential for studying fMRI response patterns of subsecond dynamic range. Future work can adopt similar time‐resolved paradigm in combination with MEG or EEG and further investigates fine‐scale temporal profile of object encodings.

## CONFLICT OF INTEREST

None declared.

## AUTHORS' CONTRIBUTIONS

J.L., B.G., and M.M designed the study. J.L. and L.C. collected the data. J.L., B.G., and H.H. analyzed the data. J.L. and B.G. wrote the first draft of the article. J.L., B.G., and M.M have edited and revised the manuscript, and all authors have approved the final version of the manuscript.

## Data Availability

All data are available to the neuroimaging research community in the public domain (https://openneuro.org/datasets/ds002788). This data sharing complies with the requirements of South China Normal University and the National Natural Science Foundation of China.
